# Recombinant thrombomodulin for secondary thrombotic thrombocytopenic purpura

**DOI:** 10.1097/MD.0000000000003712

**Published:** 2016-06-17

**Authors:** Kensuke Nakamura, Ryota Inokuchi, Takahiro Hiruma, Kazuma Ohshima, Tomohiro Sonoo, Kurato Tokunaga, Kent Doi, Susumu Nakajima

**Affiliations:** a Department of Emergency and Critical Care Medicine, Hitachi General Hospital, Hitachi, Ibaraki; b Department of Emergency and General Medicine, JR General Hospital, Sibuya-ku, Tokyo; c Department of Emergency and Critical Care Medicine, The University of Tokyo Hospital, Tokyo, Japan.

**Keywords:** disseminated intravascular coagulation, platelet, recombinant thrombomodulin, secondary thrombotic thrombocytopenic purpura, thrombotic thrombocytopenic purpura

## Abstract

In the pathogenesis of thrombotic thrombocytopenic purpura (TTP), reductions in the enzyme activity of ADAMTS13, which cuts ultralarge von Willebrand multimers, generates shear stress on the microvascular endothelium, leading to platelet aggregation and the formation of a thrombus. ADAMTS13 activity is markedly decreased in typical TTP, but is only mildly reduced in secondary TTP, which concomitantly develops with primary disease. The latter develops with septic disseminated intravascular coagulation (DIC) and often causes organ failure. Recombinant thrombomodulin (rTM) is a drug that is used to treat DIC and may also remit TTP because it improves vascular endothelial dysfunction. Therefore, we herein investigated the efficacy of rTM in patients treated for the pathology of secondary TTP. Patients who were admitted to the Emergency and Critical Care Center of our hospital and met the following conditions were extracted and retrospectively analyzed: hemolytic anemia accompanied by fragmented red blood cells (Hb < 12 g/dL or lower); thrombocytopenia (<100 × 10^3^/μL); and ADAMTS13 activity <50%. Sixteen patients were included and accompanied by Kidney Disease: Improving Global Outcomes (KDIGO) stage 2 or more severe nephropathy and DIC. Eleven and 5 patients treated with and without rTM (the rTM and non-rTM treatment groups, respectively) were compared, and no significant difference was noted in their basic characteristics, such as background disease and severity. No significant difference was observed in survival rates; however, the platelet count, which is an important outcome of treatments for TTP, significantly increased in the rTM treatment group: 3.3 ± 2.6→11.3 ± 14.6 versus 3.5 ± 3.7→5.7 ± 3.9 (×1000/μL) (*P* = 0.034). Thrombotic thrombocytopenic purpura originally requires invasive treatments and its prognosis is not favorable. Blood thrombomodulin levels also markedly increase due to vascular endothelial dysfunction, whereas rTM alleviates vascular endothelial dysfunction in TTP patients with high blood TM levels, suggesting the importance of administering rTM. Thus, rTM may be effective for secondary TTP and may be adopted as adjuvant therapy.

## 1 Introduction

In thrombotic thrombocytopenic purpura (TTP) or thrombotic microangiopathy (TMA), the enzyme activity of ADAMTS13 that cuts ultralarge von Willebrand multimers (UL-VWFM) is reduced. The resulting excess UL-VWFM induces platelet aggregation, which leads to severe microvascular endothelial disorder and thrombosis.^[1]^ The characteristic symptoms of TTP are hemolytic anemia accompanied by fragmented red blood cells, thrombocytopenia, and thrombus-induced organopathy, mainly nephropathy. In typical TTP, an ADAMTS13 inhibitor appears in the circulation and markedly decreases ADAMTS13.

Accordingly, fundamental treatments for TTP involve removal of the ADAMTS13 inhibitor by plasma exchange and supplementation with ADAMTS13 or through the administration of fresh frozen plasma (FFP). Although the survival rate of TTP patients has been improved by these treatments, the mortality rate is still nearly 20%, and its prognosis is not favorable.^[2]^ Therefore, further treatments are needed for TTP. The efficacies of steroids^[3]^ and rituximab^[4]^ have been demonstrated; however, these drugs cause severe immunosuppression and its adverse reactions may be fatal in some cases.

Another pathology termed secondary TTP is characterized by excess UL-VWFM inducing platelet aggregation and promoting vascular endothelial dysfunction due to inflammation with a decrease in ADAMTS13, but not the ADAMTS13 inhibitor,^[5,6]^ being mild.^[7]^ Regarding the diagnosis of TTP/TMA, cases complicated by disseminated intravascular coagulation (DIC) have been excluded in some studies,^[5]^ whereas the disease has been diagnosed regardless of the presence or absence of DIC in others.^[8,9]^ Organopathy is severe in DIC accompanied by reductions in ADAMTS13 activity, similar to that in TTP, and also shares symptoms with TTP.^[10]^ The pathology of TTP also occurs in DIC, such as a decrease in ADAMTS13, platelet aggregation, and thrombus formation due to excess UL-VWFM. Moreover, the mortality rate of patients with secondary TTP is 18% to 56%, which is higher than that of typical TTP patients, and the efficacy of plasma exchange is low.^[11–13]^ Thus, effective treatments are more urgently needed for secondary TTP.

Recombinant thrombomodulin (rTM) is an anticoagulant that is used to treat DIC and expected to alleviate vascular endothelial dysfunction.^[14]^ Therefore, theoretically, rTM may be effective as a treatment for TTP.^[15,16]^ We previously described the successful remission of secondary TTP by rTM monotherapy.^[17]^ Since rTM causes fewer adverse reactions without invasiveness and immunosuppression, unlike existing TTP treatments, it may serve as a therapeutic drug for TTP.

We retrospectively analyzed patients who were admitted to the intensive care unit (ICU) of the emergency and critical care center and assumed to have secondary TTP based on ADAMTS13 activity measurements, and also investigated the efficacy of the administration of rTM. Since ADAMTS13 activity levels were lower than 50% in many patients in a previous analysis of secondary TTP,^[18]^ patients with ADAMTS13 activity <50% were included.

## 2 Materials and methods

### 2.1 Subject and data analysis

Of the patients admitted to the ICU of Hitachi General Hospital Emergency and Critical Care Center and treated at the Department of Emergency and Critical Care Medicine between April 2013 and March 2015, those who met the following conditions were extracted and retrospectively analyzed: (i) hemolytic anemia accompanied by fragmented red blood cells (Hb < 12 g/dL or lower); (ii) thrombocytopenia (<100 × 10^3^/μL); and (iii) ADAMTS13 activity <50%. These inclusion criteria were prepared following the diagnostic criteria of TTP in Japan.^[18]^ In this study, hemolytic anemia was diagnosed when the following 4 findings were all satisfied: haptoglobin <20 mg/dL, lactate dehydrogenase >240 IU/L, Hb <12 g/dL, and fragmented red blood cells identified in blood smear. We selected the patients by including the patients in whom ADAMTS13 activity was <50%, and sifting them whether the other criteria had met. This study was approved by the Ethics Review Board of our hospital.

Data such as the platelet count and fibrin/fibrinogen degradation products FDP, and severity scores such as acute physiology and chronic health evaluation 2 (APACHE2) and sequential organ failure assessment (SOFA), were extracted at the time of ADAMTS13 activity measurements and subsequently analyzed.

The patients included were divided into those administered and not administered rTM at the time of ADAMTS13 activity measurements. Ricomodulin (Asahikasei Pharma, Japan) was used for rTM. rTM 12800 U is almost equal to 2 mg thrombomodulin. In Japan, 380 U/kg rTM is given to the patients with normal kidney function, and properly reduced to 130 U/kg with kidney dysfunction.

Because platelet counts reflect directly the progression of TTP and platelet recovery generally suggests the recovery from TTP condition, the platelet counts can be considered as the main outcome of this study. We also investigated the outcome of patients and analyzed the mortality rate and length of ICU stay as the secondary outcome.

### 2.2 Classification of acute kidney injury and DIC

Acute kidney injury (AKI) was evaluated as organopathy. AKI was staged using Kidney Disease: Improving Global Outcomes (KDIGO).^[19]^ Renal recovery was defined as withdrawal from renal replacement therapy at the time of discharge from ICU.

Coagulation abnormalities were scored using the Japanese diagnostic criteria of acute phase DIC^[20]^ (Table 1). Data at the time of ADAMTS13 activity measurements were used in both evaluations. Scoring system of DIC is shown in Table 1.

**Table 1:**
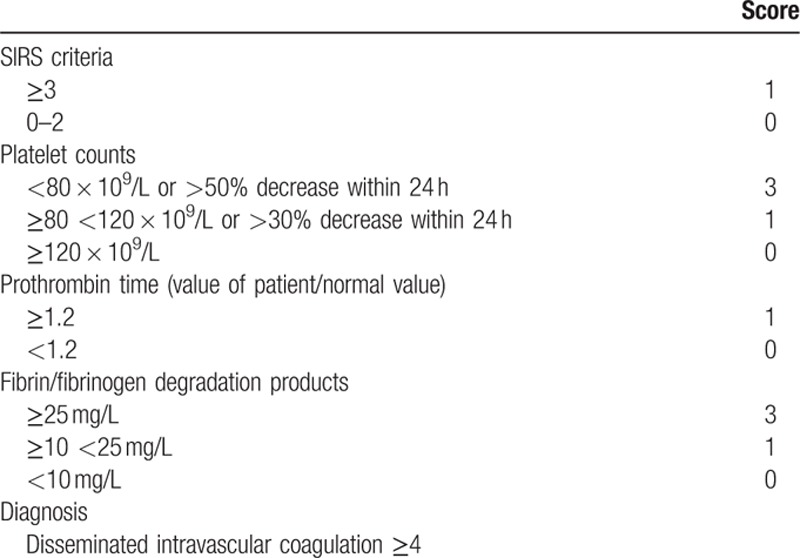
Scoring system for disseminated intravascular coagulation by the Japanese Association for Acute Medicine.

### 2.3 Statistical analysis

Differences were assessed using Student *t* tests and 1-way analysis of variance (ANOVA). Statistical analyses were performed using software (JMP 10; SAS Institute Inc.). Results were expressed as mean ± standard deviation (SD). *P* < 0.05 was inferred as significant.

## 3 Results

Eight males and 8 females were included. Their ages were 75.0 ± 11.7 years. rTM was administered in 11 patients at the time of ADAMTS13 activity measurements. The rTM dose was 12,800 U/d in all patients because nephropathy, as described below, was observed. No other anticoagulant was used until day 7, at which time analyses were performed.

The basic characteristics of our subjects are shown in Table 2. No laterality was noted in age or the sex ratio between the rTM and non-rTM treatment groups. Since the subjects were patients admitted to the Critical Care Center ICU, the APACHE2 and SOFA scores indicated very high severity. Severity was slightly higher in the rTM treatment group, whereas no significant difference was noted in the baseline platelet count. A frequent underlying disease was infection, and some patients had autoimmune disease and malignant tumors; however, there was no transplantation-related patient.

**Table 2:**
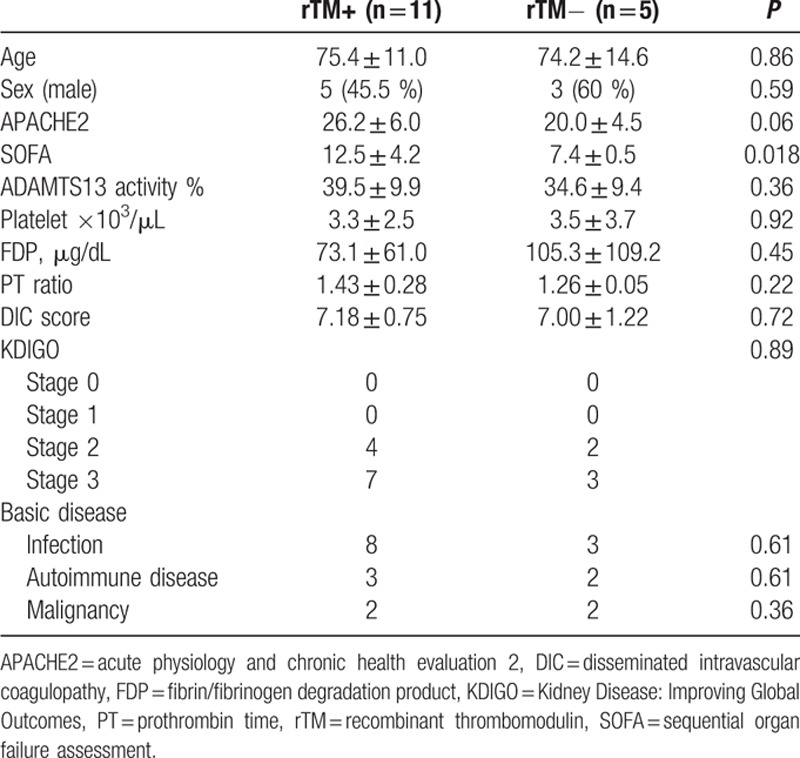
Basic characteristics at the evaluation of ADAMTS13 activity.

The ADAMTS13 activity levels were 39.5 ± 9.9% and 34.6 ± 9.4%, respectively, in the rTM and non-rTM treatment groups, and platelet counts were 3.3 ± 2.5 and 3.5 ± 3.7 × 10^3^/μL, respectively; hemolytic anemia accompanied by fragmented red blood cells was observed in both groups. The ADAMTS13 inhibitor was negative in all patients, and no marked decrease in ADAMTS13 activity levels to lower than the detection sensitivity was noted in any patient. All patients met the Japanese diagnostic criteria of acute-phase DIC. Plasma exchange was only performed on 1 patient in each group.

Regarding organopathy, which is a symptom of TTP, nephropathy was evaluated using KDIGO. Stage 2 or more severe AKI was noted in all patients. Although disturbance in consciousness is an important symptom of TTP, it could not be accurately evaluated because all patients required sedation for artificial respiration. Basic characteristics, treatments, and outcome of each patient are shown in Table 3.

**Table 3:**
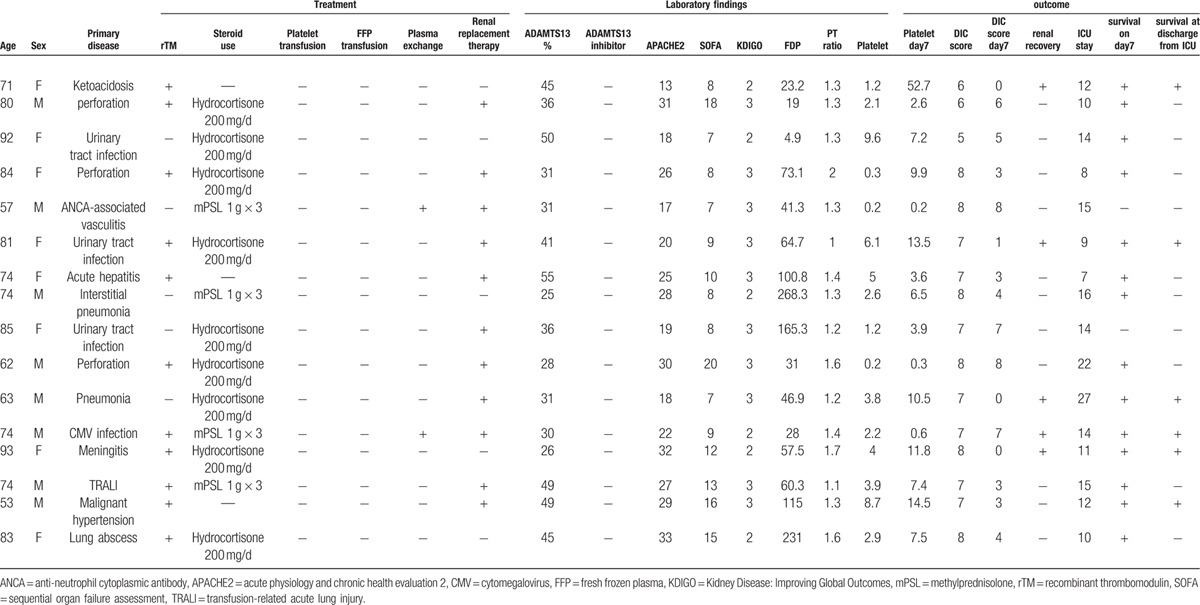
Basic characteristics, treatments, and outcome of each patient.

The main outcomes are shown in Table 4. The administration of rTM did not cause any serious adverse event, such as hemorrhage. rTM is a therapeutic drug for DIC and improves abnormal hypercoagulation. Accordingly, the DIC score was significantly improved in the rTM treatment group (*P* = 0.0004). The platelet count, which is considered to be the main outcome of TTP, was significantly improved in the rTM treatment group (rTM+: 3.3 ± 2.6 → 11.3 ± 14.6; rTM−: 3.5 ± 3.7 → 5.7 ± 3.9 × 10^3^/μ∗ (*P* = 0.034)). During the period from days 1 to 7, no platelet transfusion was performed in either group in consideration of the TTP pathology. Mortality was showed very high, because very severe patients were included in this study. It suggested that secondary TTP in critical care patients indicate very severe and poor prognosis. Renal recovery rate was higher in rTM groups; however, there was no significance. No significant differences were also observed in mortality rates at the time of discharge from the ICU; however, the length of the ICU stay was 11.8 ± 1.4 days in the rTM treatment group and 17.2 ± 2.1 days in the non-rTM treatment group, showing a significant improvement (*P* = 0.047).

**Table 4:**
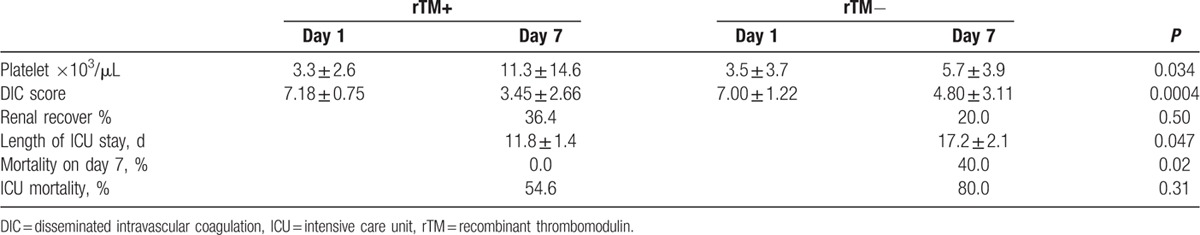
Outcome.

## 4 Discussion

To the best of our knowledge, this is the first study to examine the effects of rTM on secondary TTP. Patients assumed to have very severe secondary TTP were retrospectively analyzed in our study. rTM significantly increased the platelet count and recovered the TTP condition.

It currently remains controversial whether the pathology complicated by DIC may be included in TTP. The complication of DIC was excluded to homogenously include diseases in some studies examining the treatment of TTP,^[5,18,21]^ whereas no criterion for coagulation was set in the diagnostic criteria of TTP in some countries in which the disease concept of DIC is not generalized.^[8,9]^ On the other hand, in a previous study in which septic DIC cases were collected and retrospectively analyzed, the organopathy complication rate, such as nephropathy, was significantly higher in patients with reductions in ADAMTS13 activity, and a pathology similar to that of secondary TTP occurred in the presence of DIC.^[10]^ In our study, all patients included as secondary TTP cases were complicated by DIC, and KDIGO stage 2 or more severe AKI was noted in all patients, which is an important result. Although DIC is often complicated by nephropathy, the nephropathy complication rate in DIC patients was approximately 20% in a Japanese clinical study,^[22]^ whereas that in severe DIC cases was approximately 50%, even when mild AKI cases were included.^[23]^ It is improbable that all patients develop KDIGO stage 2 or more severe AKI. Thus, the secondary TTP-like pathology may develop in DIC patients with hemolytic anemia accompanied by fragmented red blood cells and ADAMTS13 activity reductions, such as in those meeting the inclusion criteria of this study, and this pathology may concomitantly develop with DIC in severe cases.

Recombinant thrombomodulin is a therapeutic drug that is used in the treatment of DIC due to its anticoagulant effects, and was initially used clinically in Japan. Thrombomodulin is an integral membrane type 1 glycoprotein expressed on the luminal surface of vascular endothelial cells. It activates protein C when it binds to thrombin synthesized in the body,^[24]^ and induces the anticoagulant effects of protein C. Actions other than anticoagulant effects have also been described. Anti-inflammatory effects not via protein C activation, such as the adsorption of High Morbidity Group Box-1 and binding to lipopolysaccharide by lectin domain, were reported previously.^[25]^ rTM physiologically alleviates vascular endothelial dysfunction. Protein C, activated by rTM, was previously shown to be capable of alleviating vascular endothelial dysfunction.^[26]^ Moreover, rTM lectin domain directly alleviates vascular endothelial dysfunction through its various anti-inflammatory and anticoagulant effects,^[14]^ not via protein C. In TTP, in which the main pathology is vascular endothelial dysfunction induced by excess UL-VWFM, rTM is theoretically an effective drug.

Blood thrombomodulin concentrations increase to an abnormally high level in TTP, reflecting vascular endothelial dysfunction.^[27]^ Mori et al^[27]^ found that TTP became severe, and the mortality rate and incidence of nephropathy increased with elevations in blood TM levels; however, blood thrombomodulin detected in that study reflected the degradation products of thrombomodulin expressed in the vascular endothelium and did not exhibit the biological activity described above.^[28]^ Accordingly, supplementation with rTM, which has physiological activity and alleviates vascular endothelial dysfunction, may be more beneficial for patients with marked vascular endothelial dysfunction and high blood TM levels, and may also be more effective for TTP.

Various treatments have been proposed for TTP, including plasma exchange. Although the prognosis of this disease has improved, intractable TTP cases remain.^[11]^ Furthermore, the current treatments including plasma exchange are invasive and cause adverse reactions. Since rTM was reported to be relatively minimally invasive drug,^[22]^ its applicability as adjuvant therapy is strongly expected.

There were several limitations to this study. Since this was not a prospective randomized controlled trial, the use of rTM cannot be strongly recommended based on the results obtained herein. It is difficult to perform a prospective clinical study because few patients have the pathology of secondary TTP and severe cases have a poor prognosis, as observed with the patients of this study. Other confounding factors may have been present between the rTM+ and rTM− groups.

## 5 Conclusions

The efficacy of rTM for secondary TTP was retrospectively analyzed. The main outcome of TTP, the platelet count, was significantly increased by rTM, suggesting that rTM is effective for the treatment of TTP.
